# Genetic Patterns of Oral Cavity Microbiome in Patients with Sickle Cell Disease

**DOI:** 10.3390/ijms25168570

**Published:** 2024-08-06

**Authors:** Faisal Al-Sarraj, Raed Albiheyri, Mohammed Qari, Mohammed Alotaibi, Majid Al-Zahrani, Yasir Anwar, Mashail A. Alghamdi, Nada M. Nass, Thamer Bouback, Ibrahim Alotibi, Osman Radhwi, Bayan H. Sajer, Alya Redhwan, Mohammed A. Al-Matary, Enas A. Almanzalawi, Hazem S. Elshafie

**Affiliations:** 1 Department of Biological Sciences, Faculty of Science, King Abdulaziz University, Jeddah 21589, Saudi Arabia; ralbiheyri@kau.edu.sa (R.A.); yanwarulhaq@kau.edu.sa (Y.A.); mamalgamdi2@kau.edu.sa (M.A.A.); nmnass@kau.edu.sa (N.M.N.); tbouback@kau.edu.sa (T.B.); bsajer@kau.edu.sa (B.H.S.); m.matri777@gmail.com (M.A.A.-M.); ealmanazalawy@kau.edu.sa (E.A.A.); 2 Centre of Excellence in BioNanoscience Research, King Abdulaziz University, Jeddah 21589, Saudi Arabia; 3 Hematology Department, Faculty of Medicine, King Abdulaziz University, Jeddah 21589, Saudi Arabia; drqari200@gmail.com (M.Q.); oradhwi@kau.edu.sa (O.R.); 4 Hematology Research Unit, King Fahd Medical Research Center, King Abdulaziz University, Jeddah 21589, Saudi Arabia; 5 Biological Science Department, College of Science and Art, King Abdulaziz University, Rabigh 21991, Saudi Arabia; maalzahrani4@kau.edu.sa; 6 Immunology Unit, King Fahd Medical Research Centre, King Abdulaziz University, Jeddah 21589, Saudi Arabia; 7 Princess Dr. Najla Bint Saud Al-Saud Center for Excellence Research in Biotechnology, King Abdulaziz University, Jeddah 21589, Saudi Arabia; 8 Health Information Technology Department, Applied College, King Abdulaziz University, Jeddah 21589, Saudi Arabia; ialotibi@kau.edu.sa; 9 Department of Health, College of Health and Rehabilitation Sciences, Princess Nourah Bint Abdulrahman University, Riyadh 11671, Saudi Arabia; amredhwan@pnu.edu.sa; 10 Department of Animal Production, Faculty of Agriculture, Sana’a University, Sana’a P.O. Box 1247, Yemen; 11 Department of Agricultural, Forestry, Food and Environmental Sciences (DAFE), University of Basilicata, Via dell’Ateneo Lucano 10, 85100 Potenza, Italy

**Keywords:** systemic diseases, sickle cell anemia, next-generation sequencing, metagenomics

## Abstract

The Middle Eastern prevalence of sickle cell anemia, a genetic disorder that affects red blood cells, necessitates additional research. On a molecular level, we sought to identify and sort the oral microbiota of healthy individuals and those with sickle cell anemia. Furthermore, it is crucial to comprehend how changes in the genetic makeup of the oral microbiota impact the state of sickle cell anemia. Using next-generation sequencing, the 16S rRNA amplicon was examined using saliva samples from 36 individuals with sickle cell anemia and healthy individuals. These samples were obtained from sickle cell anemia patients (18 samples) and healthy control participants (controls, 18 samples). Various analyses are conducted using bioinformatic techniques to identify distinct species and their relative abundance. *Streptococcus*, followed by *Fusobacterium nucleatum*, *Prevotella*, and *Veillonella* were the most prevalent genera of bacteria in the saliva of the SCA and non-SCA individuals according to our findings. *Rothia mucilaginosa*, *Prevotella scoposa*, and *Veillonella dispar* species were the dominant species in both sickle cell anemia and non-sickle cell anemia subjects. *Streptococcus salivarius*, *Actinomyces graevenitzii*, *Actinomyces odontolyticus*, and *Actinomyces georgiae* spp. were the most prevalent bacterial spp. in the studied SCA cases. The sequencing of the 16S rRNA gene yielded relative abundance values that were visualized through a heatmap analysis. Alterations in the oral microflora’s constitution can significantly affect the susceptibility of sickle cell anemia patients to develop more severe health complications. Salivary diagnosis is a potential tool for predicting and preventing oral microbiome-related diseases in the future.

## 1. Introduction

Sickle cell disease (SCD) is one of the most prevalent hereditary and congenital blood disorders worldwide [1,2]. It is caused by the polymerization of two mutant sickle β-globin subunits, resulting in the crescent or sickled form of erythrocytes [3]. Human hemoglobin production is regulated by a pair of inherited genes [4]. SCD has several genotypes, leading to significant diversity in absolute hemoglobin levels, known as hemoglobinopathies [3,5,6]. Sickle cell anemia (SCA), a specific form of SCD, is caused by the inheritance of two sickle genes, one from each parent [7,8].

Comprehensive studies have focused on the molecular mechanisms of fetal hemoglobin induction to mitigate the negative consequences in patients with SCA and other β-hemoglobinopathies [9]. SCD genotypes are classified as severe or mild depending on the type of symptoms and prognosis. During routine patient care, researchers have investigated potential clinical biomarkers according to genotype and treatment categories to monitor treatment progress [10,11,12].

Every year, between 300,000 and 400,000 newborns with SCA are born worldwide, with tens of thousands showing the most severe clinical phenotype of SCD due to homozygosity for the hemoglobin S form [13]. Sub-Saharan Africa has the highest prevalence of SCD. Approximately 1000 children with SCD are born in Africa daily, with more than 500 dying before the age of five [14]. Various locations in Côte d’Ivoire, Egypt, Lake Chad, Sudan, Lake Victoria, the coast of Kenya, Tanzania, Mozambique, and the east coast of Madagascar are projected to have an HbS allele frequency between 7.5% and 12.5% [15].

In the United States, about one in twelve African Americans carries the SCD mutation, and one in every 500 African Americans has the condition. Each year, one out of every 16,300 Hispanic-American neonates is born with SCA [16]. SCD is also prevalent in Saudi Arabia, where 0.26% of the population has the disease and 4.2% carry the sickle cell trait. The eastern province has the highest prevalence, with 1.2% of the people having sickle cell disease and 17% carrying the gene. The Saudi Premarital Screening Program (SPSP) reports a prevalence of 0.38/1000 for sickle cell disease (SCD) and 5/1000 for sickle cell trait (SCT) [17].

SCD originated in Africa in the HBB gene encoding the β-globin subunit of hemoglobin [18]. Studies estimate that 100,000 Americans suffer from sickle cell disease, with about one in every 500 African American babies born in the US having sickle cell anemia. While the disease is most common in Sub-Saharan Africa, it is also prevalent in the Middle East, Southeast Asia, and the Mediterranean [1]. Approximately 5 to 7 percent of the world’s population carries a defective hemoglobin gene. SCD affects one in fifty West Africans and is the most common genetic illness in the UK and France [19].

The oral cavity hosts a diverse group of microorganisms that live together and help each other [20,21], although some of them can be pathogenic [20]. Microorganisms that have evolved and adapted to live in this niche have colonized the oral cavity [22]. This unique and complex group of microbes forms various biofilms on all dental and mucosal surfaces [23]. According to Marsh and Zaura [24], the mouth contains around 700 different types of bacteria, fungi, viruses, archaea, and protozoa.

It is well known that sickle cell anemia (SCA) can manifest in the mouth. A study by de Carvalho et al. [25] suggests a link between SCA and jaw changes due to oral manifestations. This link is explained by the fact that systemic sclerosis affects many body parts, including the teeth, and many tooth opacities are associated with the development of dental and bone tissues, resulting in changes to the teeth [25,26,27]. Furthermore, some reports have revealed that dental caries, a multifactorial disease, is associated with SCD [28,29,30,31,32,33]. This association is promoted by various factors, such as microbial biofilm development, sugar intake, and saliva diffusion processes [34,35]. Dental cavities, changes in the oral microbiome, and other illnesses are brought on by oral microorganisms and their human hosts [36]. This study aims to examine the oral microbiota of individuals with SCA and compare it to that of a healthy control group.

## 2. Results

Nine female healthy subjects (mean age SD: 27.61 years, range: 18–47 years), nine male healthy subjects (mean age SD: 25.46 years, range: 21–48 years), eight female SCA patients (mean age SD: 33.37 years, range: 22–49 years), and ten male SCA patients (mean age SD: 19.4 years, range: 11–40 years) all gave saliva samples. Notably, all of the patients had sickle cell disease, even though they were generally healthy. Also, all of the people who took part in the study were receiving regular medical exams at the time that samples were collected. At first, the subjects were asked to sign the consent-to-agreement form. The people were then told to put saliva into a 5 mL tube (up to 2 mL). The samples were then taken right away to the Department of Science in the Faculty of Biology at King Abdulaziz University. There, they were put in a freezer set to 80 degrees Celsius so that they could be studied further.

The 16S Metagenomics Analysis tool [37] was used to look at the sequenced PCR regions in the 36 samples, and the tag number of the 16S-V3-V4 sequenced region was 5000. In order to do taxonomic assignment [38], amplicon sequences were grouped together and put together into operational taxonomical units using closed reference clustering. The number of reads for the samples per OUT ranged between (312–477). The PCR of the investigated samples was processed with high throughput sequencing, and the OUT taxonomic data among the study subjects showed that the OTU1 rank has the highest abundant rate (487772) read sequences across the samples; the most abundant sequences were for the bacterial spp.: *Firmicutes*; *Bacilli*; *Lactobacillales*; *Strptococcaceae*; *Streptococcus*. While the OTU41 rank has the lowest abundant rate (20531) read sequences across the samples, the most abundant sequences were to the bacterial spp.: *Firmicutes*; *Negativicutes*; *Selenomonadales*; *Veillonellaceae*; *Veillonella*; *Veillonella atypica*.

**Figure 1:**
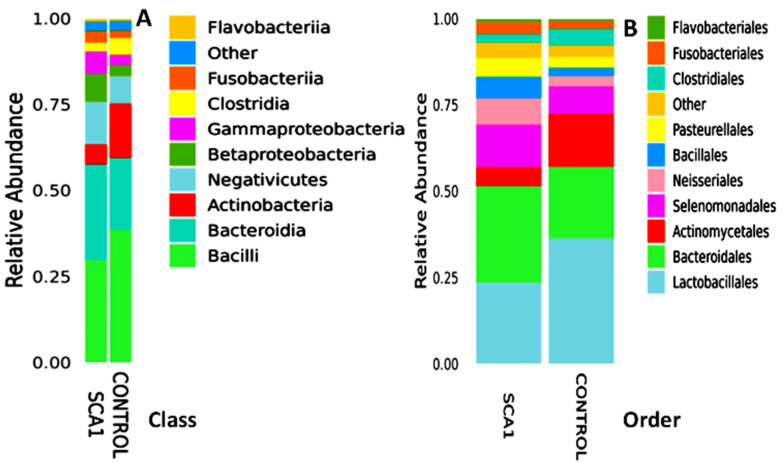
Abundance bar plot shows the composition and proportion of bacterial species at the genus (**A**) and order (**B**) levels in the SCA and healthy groups. Species will be classified into ‘others’ if their relative abundance is less than 0.5%.

A total of 346 species of bacteria were identified from the saliva samples of the healthy and SCA people in this study. The most common class and order were used for the bar plots and figures that follow. The relative abundance bar plot at the class and order levels can be seen in Figure 1. As you can see from the species abundance bar plot, the bacteria in each sample or group of SCA patients and healthy controls are made up of different types and amounts. A species will be labeled as ‘others’ if its relative abundance is less than 0.5%. Negativicutes and betaproteobacteriai were the next most common types of bacteria in both the SCA and healthy samples. But there are more bacilli and negativicutes in the saliva samples of the control groups than in the saliva samples of the SCA patients. On the order level of the study subjects, the most common orders were bacteroidales and lactobacillales, followed by selemonadales and neisseriales.

**Figure 2:**
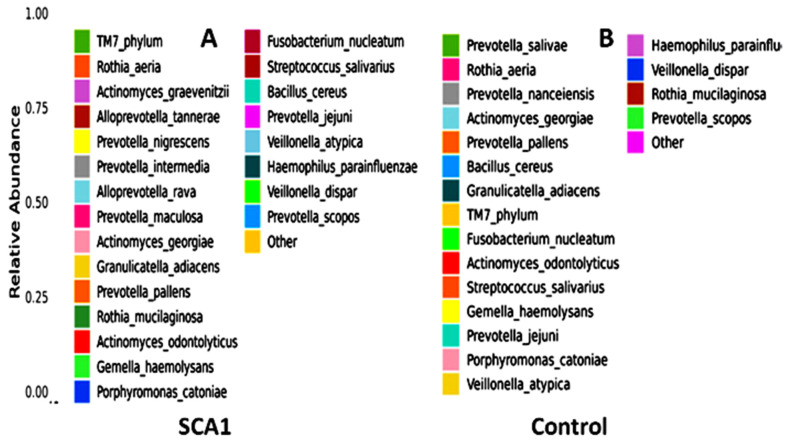
Abundance bar plot shows the 23 most commonly found bacterial species in the SCA group (**A**) and the 19 most commonly found species in the healthy control group (**B**). Species will be classified into ‘others’ if their relative abundance is less than 0.5%.

Figure 2 shows that the most common types of bacteria in the saliva of the SCA patients and healthy people are from the Lactobacillales and Bacteroidales orders. On the other hand, there were more Lactobacillales and Actinomycetales in the control group than in the SCA group.

**Figure 3:**
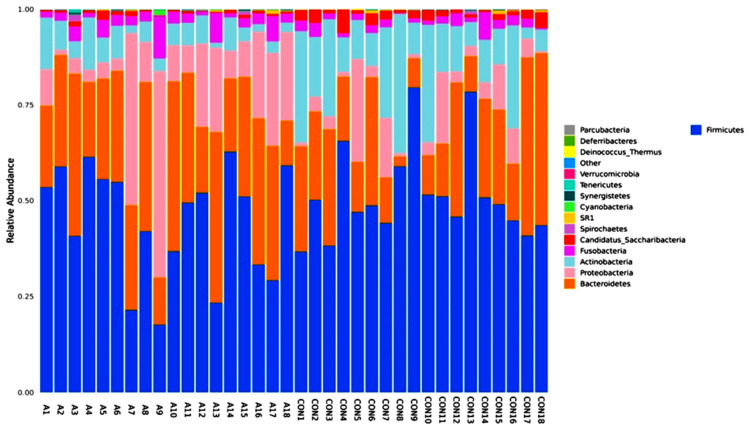
Relative abundance graph shows the composition and proportion of bacterial species at the phylum level between the individual SCA and control groups. Species will be classified into ‘others’ if their relative abundance is less than 0.5%.

**Figure 4:**
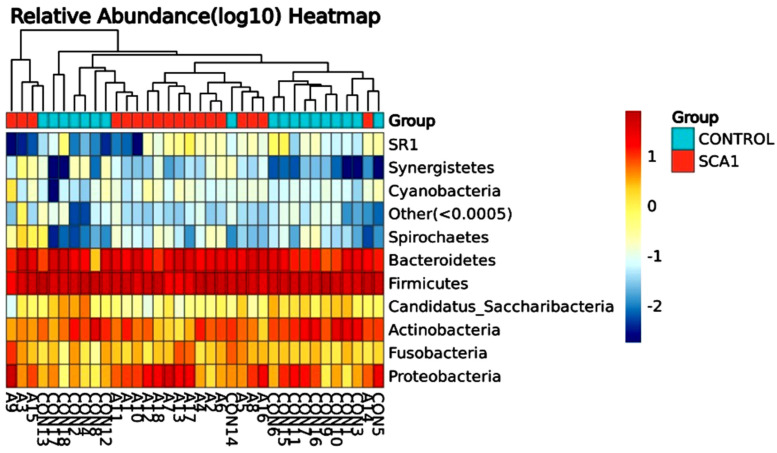
Phylum heatmap hierarchical clustering analysis showed the relative abundance values obtained by the 16S rRNA amplicon sequencing. The horizontal clusters indicate the similarity of certain phyla among the SCA and non-SCA samples.

**Figure 5:**
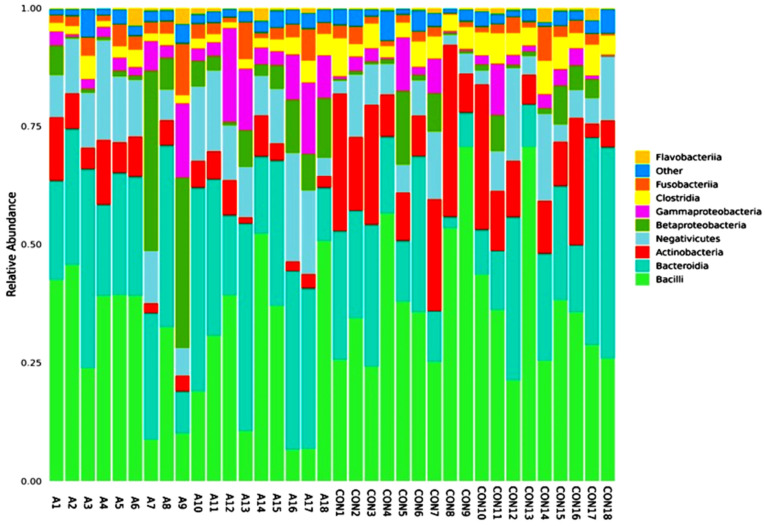
Relative abundance graph shows the composition and proportion of bacterial species at the class level between the individual SCA groups. Species will be classified into ‘others’ if their relative abundance is less than 0.5%.

**Figure 6:**
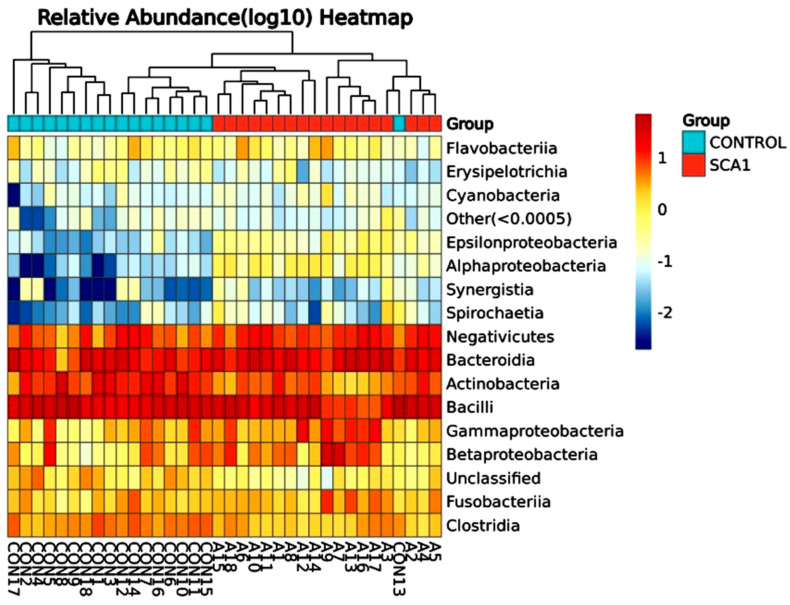
Bacterial species class heatmap hierarchical clustering analysis showed the relative abundance values obtained by the 16S rRNA amplicon sequencing. The horizontal clusters indicate the similarity of certain classes among the SCA and non-SCA samples.

**Figure 7:**
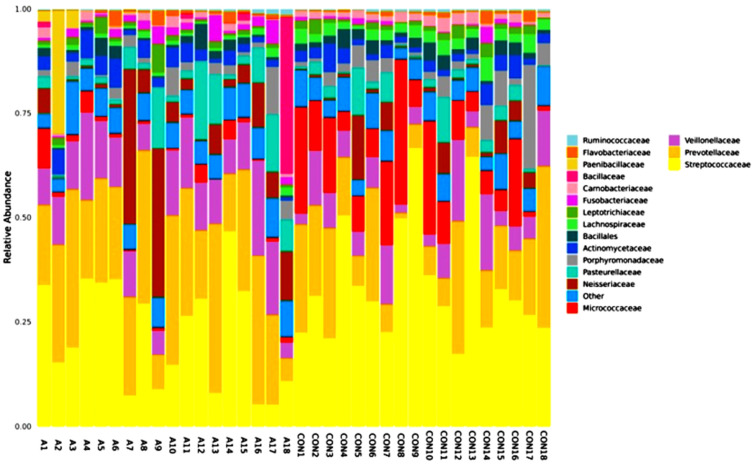
Relative abundance graph shows the composition and proportion of bacterial species at the family level between the individual SCA groups. Species will be classified into ‘others’ if their relative abundance is less than 0.5%.

**Figure 8:**
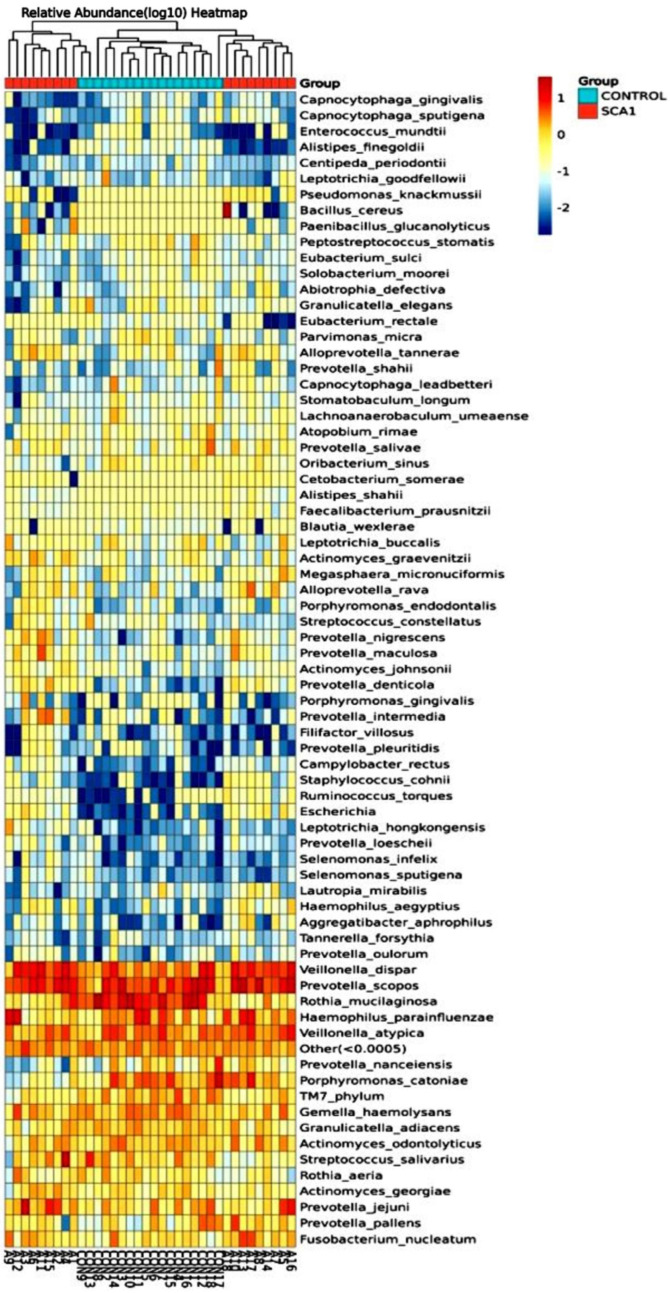
Species family heatmap hierarchical clustering analysis showed the relative abundance values obtained by the 16S rRNA amplicon sequencing. The horizontal clusters indicate the similarity of certain families among the SCA and non-SCA samples.

Figure 3 shows the differences in the amounts of oral microbiota (phylum) in the different samples. The display for phylum heatmap hierarchical clustering analysis is shown in Figure 4. The relative abundance of bacterial species at the class level is represented in Figure 5, and the heatmap hierarchical clustering analysis is displayed in Figure 6. The relative abundance of bacterial species at the family level is represented in Figure 7, and the heatmap hierarchical clustering analysis is displayed in Figure 8. The Firmicutes phylum had the most growth in this study. The Bacteroidetes phylum came in second. In addition, the number of Proteobacteria microbiota increased in both the SCA and control groups. These results are similar to those of Alyousef et al. [39], who found that the mouths of SCA patients had a very high concentration of Proteobacteria. More Firmicutes, Proteobacteria, and Actinobacteria phyla were found in the samples from the healthy people than in the samples from the SCA patients. Additionally, the samples A14 and A4 had the highest percentage of Firmicutes, while the samples A9 and A7 had the lowest concentration. The levels of the phylum Proteobacteria are the highest ever recorded in these two samples, which means that the number of these bacteria is going down. Compared to the other people in the group, A6 had very high levels of proteobacteria. There was no difference between the SCA of males and females because A9 is male and A7 is female and they have high proteobacteria content. Comparing phylum diversity between males and females did not show any differences. Deferribacteria were only found in sample A9, which is very interesting.

Firmicutes is a big group of bacteria that includes more than 250 genera, like Clostridium, Lactobacillus, Mycoplasma, Streptococcus, and Bacillus. Furthermore, lactobacilli have the metabolic power to make a lot of acid and are highly acidogenic. This has been linked to the loss of minerals in dental tissue and the formation of dental cavities. Also, Lactobacilli can live in environments with very low pH [40].

**Figure 9:**
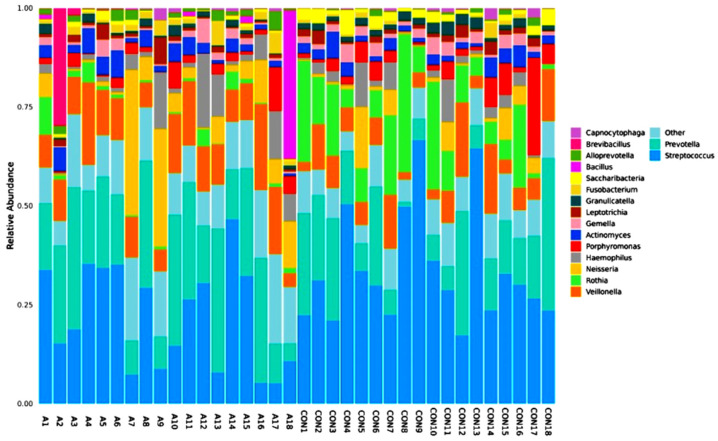
Relative abundance graph shows the composition and proportion of bacterial species at the genus level between the individual SCA groups. Species will be classified into ‘others’ if their relative abundance is less than 0.5%.

**Figure 10:**
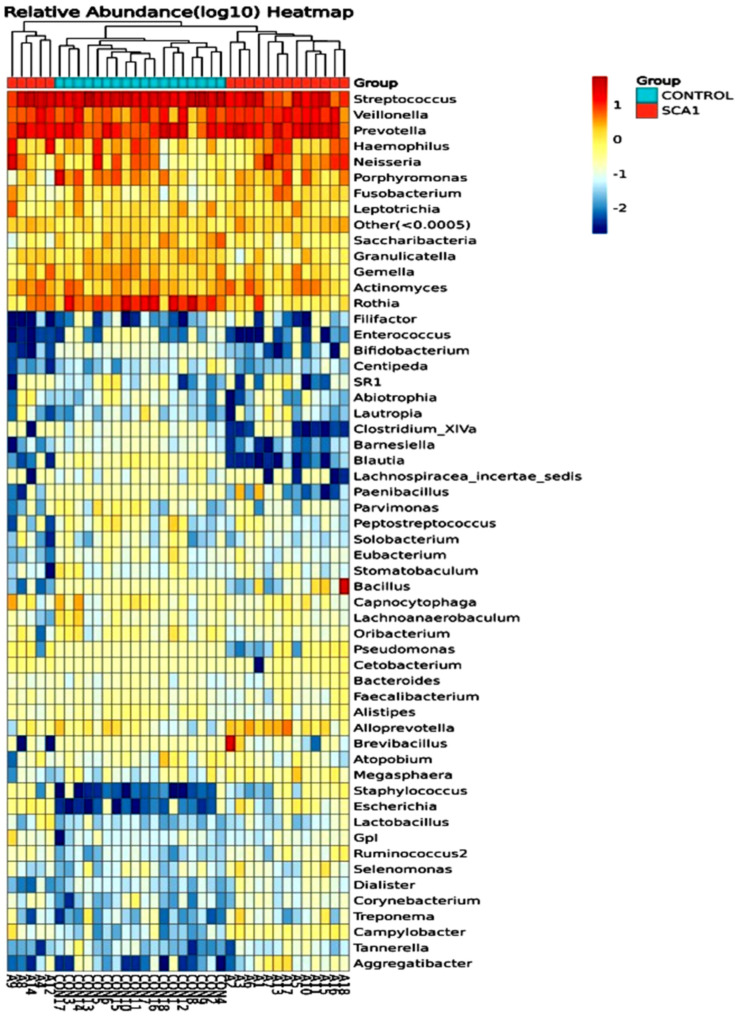
Species genus heatmap hierarchical clustering analysis showed the relative abundance values obtained by the 16S rRNA amplicon sequencing. The horizontal clusters indicate the similarity of certain genera among the SCA and non-SCA samples.

Figure 9 shows the number of microbes at the genus level in the people with SCA and healthy controls, and the heatmap is shown in Figure 10. Streptococcus is the most common genus, and Prevotella is the next most common. Streptococcus is more common in the control samples than in the SCA group subjects. On the other hand, Prevotella is more common in the SCA disease subjects than in the control samples. Additionally, the samples CON9, CON13, CON8, CON4, and A14 had the highest amounts of Streptococcus. These were followed by the samples CON10, CON5, CON15, A1, and A15. The samples A7, A13, A16, and A17 had the lowest amounts of streptococcus. The graph also shows that the genus Prevotella is the second most common in both the SCA and control subjects. This is because it is one of the largest genera, with about 50 species, most of which have been grown in gardens [41]. The samples A18 and A1 from the people with SCA had the most bacillus of any of the others. The graph also shows that all of the samples had very low amounts of Sacchari bacteria, except for the samples A7 and A9, which had a significant amount of this genus. The saliva samples of Sacchari bacteria genera, which used to be called TM7, are part of the Candidate Phyla Radiation and can be found in the oral microbiota of humans. Streptococcus and Veillonella were found to be the most common genera in saliva after a tooth had been erupted and in plaque [42].

Streptococcus bacteria are thought to be among the first to colonize the mouth. They are picked up soon after birth and are very important to the development of the oral microbiome. They make sticky molecules that let them settle in different oral tissues and spaces. They are also very good at breaking down carbohydrates and making acids as byproducts. There is a direct link between acid uric species like Streptococcus mutants that make the mouth too acidic and dental cavities and diseases. Dental caries and periodontitis are both linked to dental plaque bacteria and the metabolic processes they carry out. A recent study looked at the microbiomes that live on top of gingival plaque in cavities and healthy areas. It found that acid-producing and acid uric species like *S. mutans*, Scardovia wiggsiae, Parascardoviadenticolens, and *Lactobacillus salivarius* were different in kids who had different levels of cavities. Even so, the Streptococcus genus is very important in maintaining oral health and disease because it can change its metabolism, colonize many oral surfaces and niches, and make substances that stop other bacteria from doing their job [43].

**Figure 11:**
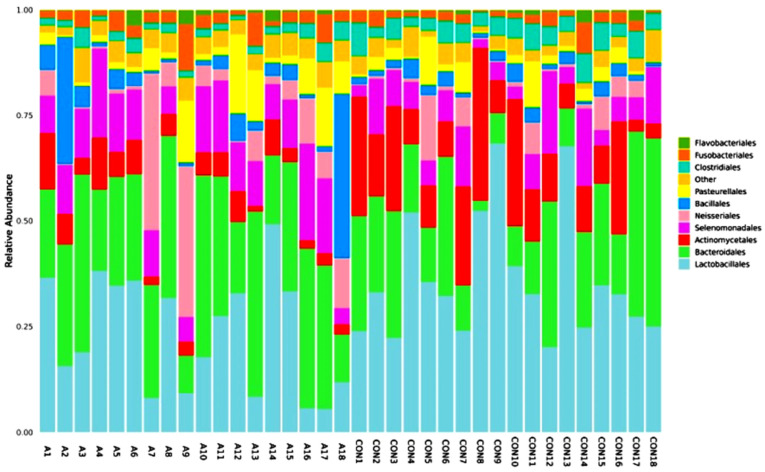
Relative abundance graph shows the composition and proportion of bacterial species at the order level between the individual SCA groups. Species will be classified into ‘others’ if their relative abundance is less than 0.5%.

**Figure 12:**
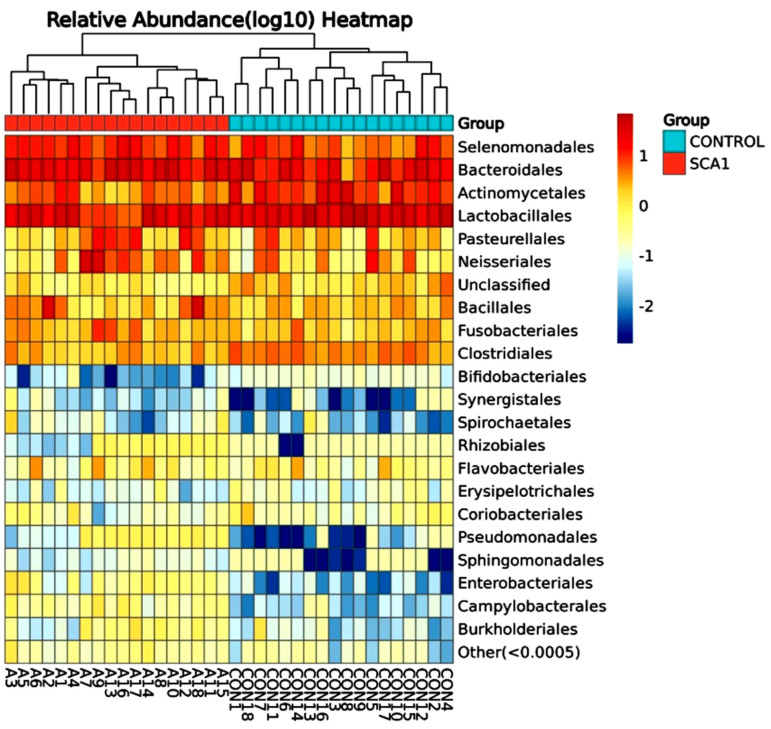
Species order heatmap hierarchical clustering analysis showed the relative abundance values obtained by the 16S rRNA amplicon sequencing. The horizontal clusters indicate the similarity of certain orders among the SCA and non-SCA samples.

The number of microbes at the order level in the individuals with SCA and healthy controls is shown in Figure 11 and as a heatmap in Figure 12. Both the SCA and non-SCA saliva samples contained significant amounts of bacteriophages and lactobacillales. However, lactobacillales were more prevalent in the non-SCA saliva samples than in SCA saliva samples, whereas bacteriophages were more common in the saliva of the SCA patients than in the control subjects.

The samples with the most bacterial orders were A3, A13, A10, CON17, and CON18, while those with the least were CON8, CON10, and A9. Notably, the samples A7 and A9 had the highest amounts of the Neisseriales order. Bacillales were also present in large numbers in the samples A18 and A2. More of the Actinomycetales order was found in the healthy, non-SCA saliva samples compared to the SCA samples, with the highest levels observed in CON8, CON10, CON1, CON7, and CON16. The order Pasteurellales was not very common in any of the samples, and all the saliva samples from both the SCA and non-SCA individuals had very low levels of Clostridiales.

Comparative studies showed that the oral microbiome of the male and female SCA patients did not differ significantly. Bacteroidales, important periodontal pathogens, are known to cause periodontitis and other oral diseases, as well as systemic illnesses. They can infect many areas of the body, including the mouth. A previous study found a link between the incidence of aggressive periodontitis and the number of bacterial pathogens [44].

**Figure 13:**
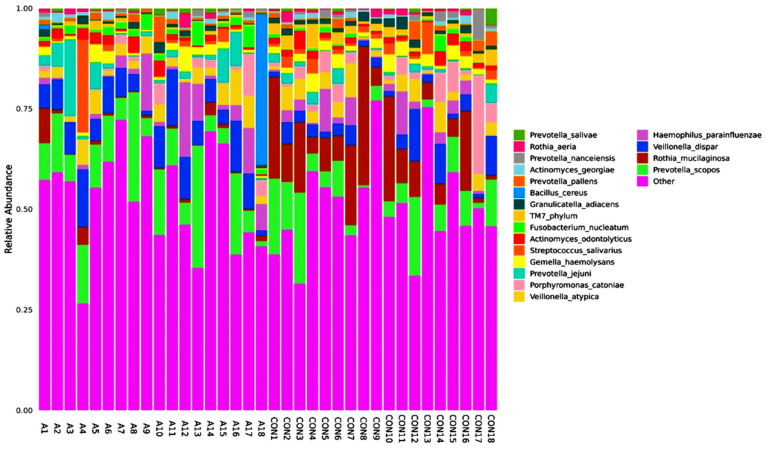
Relative abundance graph shows the composition and proportion of bacterial species at the species level between the individual SCA groups. Species will be classified into ‘others’ if their relative abundance is less than 0.5%.

**Figure 14:**
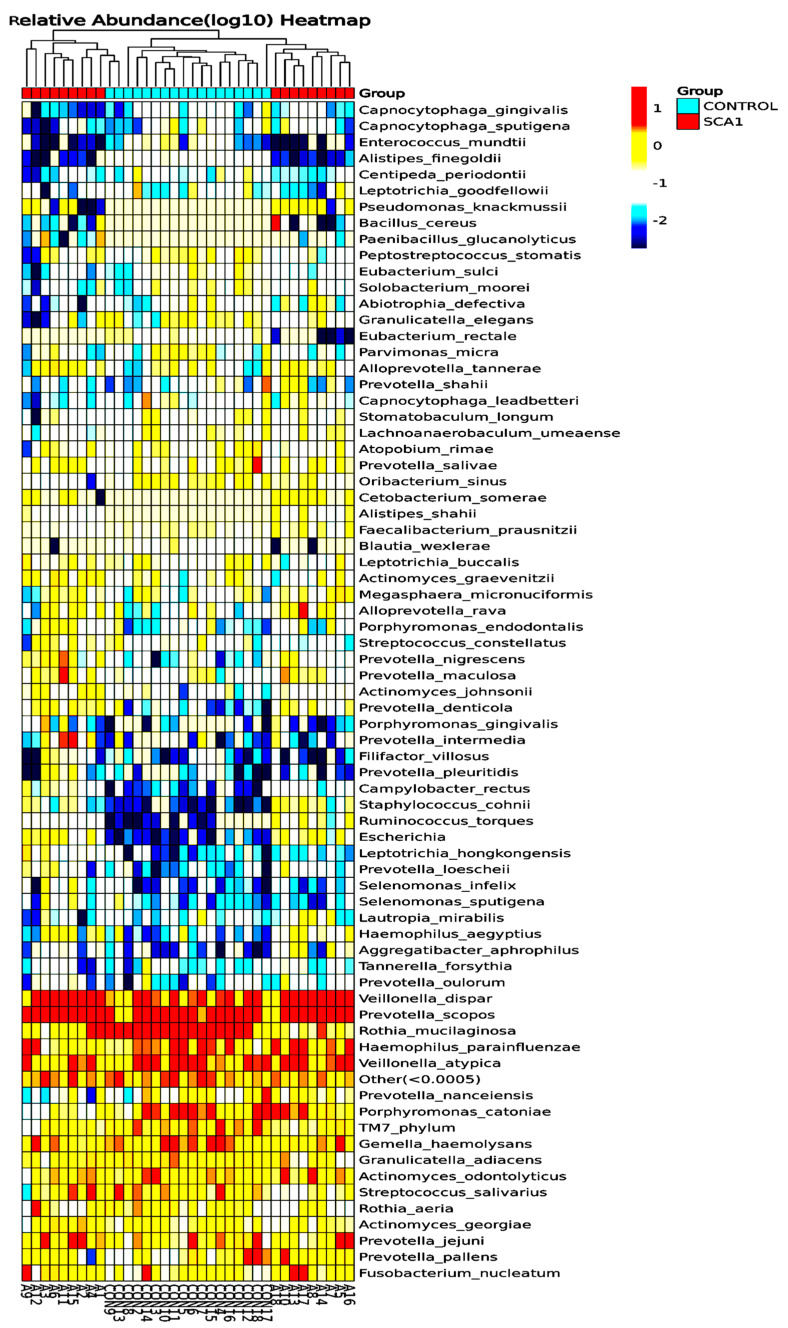
Species heatmap hierarchical clustering analysis showed the relative abundance values obtained by the 16S rRNA amplicon sequencing. The horizontal clusters indicate the similarity of certain species among the SCA and non-SCA samples.

There are more of the *Rothia mucilaginosa* species in the saliva samples from the healthy control subjects than in the saliva samples from the SCA patients. This can be seen in both the species-level abundance of microbes in the SCA patients and healthy control subjects (Figure 13) and the heatmap hierarchical clustering visualization (Figure 14). *Prevotella scopos* was found in most of the SCA and non-SCA samples. The samples A13, A8, CON3, CON12, and A16 had the highest levels of this species. *Veillonella dispar* species was next. It is worth mentioning that sample A18 had the highest concentration of Bacillus cereus species. Small amounts of *Fusobacterium nucleatum* were found in all of the samples, but sample A3 had the highest amount of Streptococcus salivarius. In the samples, Prevotella Jejuni was also found. Early on, *P. scopos*, *P. nigrescens*, and *P. pallens*, as well as other commensal anaerobic oral prevotella can be found in the oral mucosa and dental plaques. Several types of Prevotella are often found in the harmful biofilms of periodontal diseases and play a part in oral inflammatory diseases. Dental cavities can be caused by *P. intermedia* and *P. nigrescens*, while endodontic infections and other serious oral conditions can be caused by *P. denticola*. Many new species have been found in the mouth over the years, but no one knows what clinical significance they have yet. Different species and strains of the genus Prevotella have different virulence factors and other traits, like biofilm formation. For example, jejuni was first found in the jejunum of a child with celiac disease, but it can also be found in the mouth. The made-up phylogenetic tree is shown in Figure 14.

**Figure 15:**
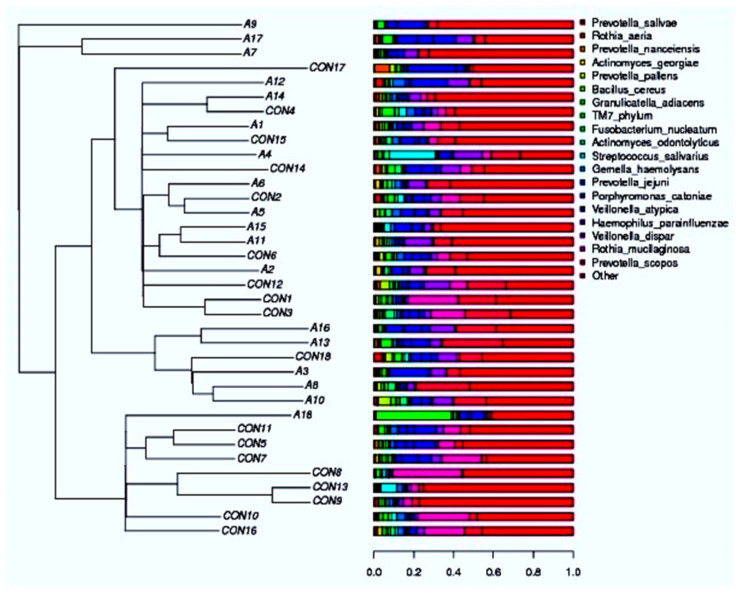
Species SCA-Control taxonomic tree of the relatively abundant bacterial isolates obtained from the next-generation sequencing for the 16S-V3-V4 regions of all the subjects.

The next-generation sequencing of the 16S-V3-V4 regions was used to identify the bacterial species in both the SCA and non-SCA saliva samples. The taxonomic classifications of all the samples were then assigned to operational taxonomic units, and differential species analysis was performed. Figure 15 shows the species SCA-Control taxonomic tree of the bacterial isolates that are fairly common. The species Rothia mucilaginosa, Prevotella scoposa, and Veillonella dispar were the most common in both the SCA and healthy people.

## 3. Discussion

This is the largest study of the microbiome ever conducted at King Abdulaziz University Hospital and the second-largest study ever performed in Saudi Arabia. This is also the second study to look into the oral microbiota of people with sickle cell anemia. The eastern and western parts of Saudi Arabia, where SCA is common, were used for this study [39]. Because dental cavities and SCA seemed to be linked, researchers used a high sex index and general health status to measure the amount of microbiota. This study adds to the collection of microbiome reference data for a population that has not been studied much. Tooth decay is brought on by an imbalance between factors that cause disease and those that protect it, which affects the processes of demineralization and remineralization. People with SCA who have dental caries problems like pulpitis, acute pain, and periapical periodontitis a lot have a low quality of life. Several studies show a link between SCA, gum problems, and cavities in the teeth. When SCA cuts off blood flow to dental tissue, it leads to pulpitis, periapical periodontitis, and a lack of the production of dental and bone tissues, which makes it more likely for infections to spread [31]. This study found that people with SCA had more Proteobacteria and Firmicutes bacteria than their peers. It was mostly Prevotellaceae and Streptococcaceae bacteria that were found in the SCA patient index group. People who have SCA have chronic inflammation, which makes it easier for facultative anaerobic microorganisms to grow because their blood flow is poor and they are in a highly inflammatory state. What the researchers found suggests are those patients have a lot of anaerobic bacteria from the phylum Prevotellaceae.

Several studies have found Prevotella species bacteria in clinical samples, suggesting that having Prevotellaceae species in the mouth may be beneficial for individuals with weak immune systems. The results of this study are consistent with the findings from studies involving young people in China and Myanmar, where children with dental cavities had more proteobacteria than those without cavities. In fact, children with dental cavities exhibited some of the most abundant oral microbiota.

In patients with SCA, *Streptococcus sanguinis* has been detected in the oral cavity. This bacterium is known to inhibit the growth of beneficial bacteria by producing hydrogen peroxide (H_2_O_2_), which can suppress the proliferation of various other microorganisms [39,45,46]. However, the presence of *S. sanguinis* alone is insufficient to form cariogenic biofilms, which are responsible for tooth decay.

This study enhances our understanding of the oral microbiota in SCA patients, a population that has not been extensively studied. The oral microbiota in individuals with SCA differs from that of healthy individuals, showing higher levels of facultative anaerobic bacteria, including those from the *Proteobacteria* and *Firmicutes* phyla. Notably, the SCA patient group exhibited a predominance of bacteria from the *Prevotellaceae* and *Streptococcaceae* families. Further research is necessary to elucidate the roles of these bacteria in the development of dental cavities and periodontal diseases in SCA patients and to develop targeted treatments [47].

## 4. Materials and Methods

### 4.1. Collection of Patient Samples

At King Abdulaziz Hospital in Jeddah, Saudi Arabia, oral samples and clinical data were collected in 2022 from 18 individuals who had a homozygous S/S genotype (SCA) and 18 healthy individuals who did not have SCA. The ages of these people who were clinically diagnosed with SCA ranged from 11 to 49 years. After obtaining baseline measurements from the hospital’s records, a qualified doctor looked at each patient’s overall health. The patients were put into groups based on their gender and age. This study followed the rules set out in the Declaration of Helsinki and Good Clinical Practice [25]. It was approved by the Institutional Review Board (IRB) committees of King Abdulaziz University (Ref-570-21).

### 4.2. DNA Extraction

All the participants received a saliva DNA microbiome collection tube containing a stabilizing buffer and were instructed to spit into the funnel until the 2 mL line was reached, ensuring that no bubbles were present. Each participant’s parent or guardian shook the tubes very hard several times to make sure the sample and solution were well mixed. After that, the tubes and their contents were kept at −80 °C until the research team found bacterial DNA sequences. We looked at the oral 16S rDNA in the saliva of the SCA patients and a control group that had few decayed, missing, or filled permanent teeth. This was performed to see how many microbial communities were present (DMFT). Microbiome DNA was separated using the Genei Pure IDTM DNA Isolation Kit-Saliva (Lot No: KT1841052, GeNei^TM^, Bangalore, India) in accordance with the manufacturer’s instructions. The samples were centrifuged at 3000 rpm for 15 min at room temperature as part of the processing/lysis stage. After that, the supernatant was discarded. Subsequently, the pellet was mixed with 5 mL of Lysis Buffer I and centrifuged at 3000 rpm for 15 min at room temperature. It was mixed again with 700 L of Lysis Buffer II and 50 L of Proteinase K at a concentration of 25 mg/mL. The supernatant was thrown away. After that, the contents were moved to a brand-new 1.5 mL Eppendorf tube. It took two minutes of heating at 40 degrees Celsius to see turbidity in the Lysis Buffer II. Once the time was up, the vial was kept at 58 °C for 45 min and then spun at room temperature for 2 min at 10,000 rpm. The supernatant was put into a new, clean 1.5 mL vial, and the same amount of 95% ethanol was added and mixed well. In the Binding and Wash step, the samples were put in a collection tube and then put on the GeneiPureIDTM spin columns. At room temperature after 10,000 revolutions per minute for one minute, the flow through was thrown away. Before it was used, four volumes of absolute alcohol were mixed thoroughly with one volume of wash buffer. The new 2 mL collection tube for the spin column had 700 L of wash buffer I added to it. After being spun at 11,000 rpm for one minute at room temperature, the flow through was thrown away. The column was put in the same collection tube and spun at 10,000 rpm for two minutes to get rid of any wash buffer that was still there. After that, the collection tube was thrown away. A 1.5 mL sterile vial was used to hold the spin column. During the elution step, the right amount of elution buffer was put into a clean 1.5 mL Eppendorf tube using a pipette. The tube was then put in a dry bath that was already heated to 70 °C for 5 min. Then, 150 mL of 70 °C pre-warmed elution buffer was pipetted into the middle of the spin column. The column was left to sit at room temperature for one to two minutes, and then it was centrifuged at 10,000 rpm for one minute while it was still at room temperature. The DNA was then put into 1.5 mL Eppendorf tubes that were clean and frozen at −20 degrees Celsius.

### 4.3. DNA Purification and Concentration Measurements

A SpectraMax QuickDrop Micro-Volume Spectrophotometer was used for fluorometry to estimate the DNA concentrations and purity (Molecular Devices, San Jose, CA, USA). SpectraMax QuickDrop was also used to measure the DNA yield fluorometrically. By using the spectrophotometric measurements of absorbance ratios on a SpectraMax QuickDrop, the purity of the DNA was determined. The absorbance ratios A260/280 nm for protein contamination and A260/230 nm for salt and phenol contamination were established.

Amplification of bacterial 16S rRNA genes and sequencing

It is possible to improve the resolution of 16S rRNA profiling by adding 30 ng of a qualified DNA template and the 16S rRNA fusion primer sets 16S-V3-V4. To finish building the library, all the PCR products are cleaned up with Agencourt AM Pure XP beads (Beckman Coulter™ A63880, Beckman Coulter Life Sciences, Indianapolis, IN, USA) mixed with elution buffer, and given labels. The Agilent 2100 Bioanalyzer (Agilent Technologies, Inc, Santa Clara, CA, USA) measures both the concentration and the size distribution of a library. The qualified libraries are then ranked according to the size of their inserts.

### 4.4. Bioinformatics Analysis and Data Filtering

The first set of data is cleaned up to make a high-quality set, and the clean reads are then combined into tags and grouped into OTUs. Each operational taxonomical unit’s (OTU) representative sequence is assigned a taxonomic classification using the Ribosomal Database Project database. The OUT-profile table and the taxonomy annotation findings were subjected to numerous analyses. These comprised network, differential species analysis, alpha and beta diversity, and model prediction. The raw data go through the following filters to obtain clean, high-quality reads: (1) Reads within a 25 bp sliding window that have average Phred quality values below 20 will be cut off. (2) Remove readings that, following truncation, had lengths less than 75% of their initial lengths. (3) Eliminate readings contaminated with adapter sequences. (4) Eliminate simple reads (reads with 10 consecutive bases by default). Using internal scripts, the clean readings were compared to barcode sequences without base mismatches. The barcode sequences were eliminated from the pooled libraries (BGI Genomics Shenzhen, China) by doing this. If two paired-end reads overlap, FLASH (Fast Length Adjustment of Short Reads, v1.2.11) creates a consensus sequence. Some specifics are given below: (1) the overlap must be at least 15 base pairs long; (2) the mismatching ratio of the overlapped region must be at least 0.1.

### 4.5. OUT Clustering and Annotation

OTUs, or operational taxonomic units, are a standard way to study a taxon unit in phylogeny or population genetics research (seven taxonomic levels). To figure out how many bacteria are at each level in each sample, the sequences need to be grouped into OTUs that are 97 percent similar. In USEARCH (v7.0.1090), tags are categorized according to the operational taxonomic unit (OTU) as follows: (1). Using a 97 percent threshold, UPARSE clusters tags into OTUs, and sample sequences can be obtained for every OTU. (2). For 16S rDNA and ITS sequences, chimeras in OTU are screened and filtered by mapping to the Gold Database (v20110519); chimeras are filtered by UCHIME (v4.2.40) and UNITE (v20140703), respectively, whereas de novo chimera screening is performed on 18S rDNA sequences. (3) Using USEARCH GLOBAL, all the tags are mapped to the representative sequences of OTUs in order to generate the OTU abundance table. These are the programs SEARCH (v7.0.1090), UCHIME (v4.2.40), and BGI Genomic. Adding a taxonomic note to OTU: the RDP classifier software (v2.2) lines up OTU representative sequences with the database so that taxonomic information can be added (sequence identity is set to be 0.6). The default date for the 16S database, which includes bacteria and archaea, is 30 September 2016, and the date for the 18S fungus is 16 December 2019, which is also the default date for Silva. Change 8.2 of the ITS fungus UNITE (default). Here’s how to filter the results for annotations—the rest of the OTUs are used for more research.

### 4.6. Species Composition and Abundance

The representative OTU sequences are taxonomically analyzed using a Bayesian RDP classifier algorithm to find out what kinds of microbes are in the structure. After annotation, seven levels of abundance are found for each species (phylum, class, order, family, genus, and species). In USEARCH (v7.0.1090), tags are categorized according to the operational taxonomic unit (OTU) as follows: (1) Using a 97 percent threshold, UPARSE clusters tags into OTUs, and sample sequences can be obtained for every OTU. (2) For 16S rDNA and ITS sequences, chimeras in OTU are screened and filtered by mapping to the Gold Database (v20110519); chimeras are filtered by UCHIME (v4.2.40). (3). A species hierarchical clustering is made so that the size of the effect can be seen as a two-dimensional color map. We use the complete clustering method and the ‘Euclidean’ distance to group the samples together based on how common each species is at all seven levels. This shows which samples are similar.

### 4.7. Phylogenetic Tree Construction

The evolutionary connections between living things are shown in a phylogenetic tree. The phylogenetic trees are not facts; they are ideas. Phylogenetic trees show how species or other groups have changed over time by showing how they evolved from a group of common ancestors. Used in the building of the tree GraPhlAn (http://segatalab.cibio.unitn.it/tools/graphlan accessed on 16 February 2023) is a software that makes high-quality circular pictures of taxonomic and phylogenetic trees. GraPhlAn was also used. It focuses on making phylogenetically and taxonomically driven research representations that are clear, complete, useful, and ready for publication. Software: You can obtain Fast Tree version 2.1.3 at http://www.microbesonline.org/fasttree/ accessed on 16 February 2023. To make phylogenetic trees, the Approximately Maximum Likelihood algorithm is used. To see phylogenetic trees, R is used (v3.1.1).

### 4.8. Function Prediction

Picrust (Phylogenetic Investigation of Communities by Reconstruction of Unobserved States) predicts the functional annotations of microorganisms based on their phylogenetic profiles. Functional analysis is crucial for understanding microbial diversity from a biological perspective. By connecting species to their predicted functions, researchers can obtain comprehensive profiles of how microbial communities operate. Using the profiles of marker gene sequencing, PICRUSt2 (Phylogenetic Investigation of Communities by Reconstruction of Unobserved States) makes educated assumptions about the dynamics of microbial communities (including guessing KEGG, COG, and Meta Cyc metabolic pathways) and how many of them there are. What does ‘function’ usually mean? It means a group of genes, like the KEGG homologous gene and enzyme classification number. Most of the time, 16S rRNA gene sequencing data are used to make predictions, but other marker genes may also be used. PICRUSt2 is more accurate than PICRUSt1 and has more gene families and reference genomes stored in its database. PICRUSt2’s main tasks are the following: 1. add sequences to an existing phylogenetic tree and 2. figure out where these added OTUs should go in a reference phylogenetic tree. With hidden-state prediction (HSP), you can guess which gene family and pathway are most common in a sample. The future of the KEgg function: the KEgg function shows the following: PICRUST2 found out how many predicted KEGG functions were present in the bacterial community using the name of a functional gene as its KO ID. The abundance table for each of the three metabolic pathway levels and other relevant data is obtained by the function from the KEGG database. R and version 2.30.b of PICRUSt2 were used (v3.4.10).

## 5. Conclusions

Based on our study, individuals with sickle cell anemia (SCA) in Saudi Arabia have a higher prevalence of harmful oral microorganisms, including *Streptococcus salivarius*, *Actinomyces graevenitzii*, *Actinomyces odontolyticus*, and *Actinomyces georgiae* spp. These bacteria can contribute to oral health issues such as tooth decay and cavities, which may be exacerbated by the compromised immune systems commonly found in SCA patients, affecting both innate and adaptive immunity. Given these findings, it is crucial for individuals with SCA to undergo regular oral hygiene checkups. Our research indicates that these patients harbor a higher number of microbiota species (*Streptococcus* salivarius, *Actinomyces graevenitzii*, *Actinomyces odontolyticus*, and *Actinomyces georgiae* spp.) associated with dental cavities. However, to establish a definitive link between the oral microbiota and dental cavities in SCA patients, further well-designed longitudinal studies with larger sample sizes are needed. We recommend that individuals with SCA adhere to a stringent oral hygiene regimen, including regular dental checkups and preventive care practices such as using mouthwash, toothpaste, dental floss, and toothbrushes. Additionally, proactive steps to reduce the presence of those harmful oral bacteria should be taken. These include frequent dental visits, daily brushing, the use of mouthwash, and limiting the consumption of acidic and sugary foods.

## Data Availability

The raw data were generated at the Biological Department of King Abdulaziz University. The derived data supporting the findings of this study are available from the corresponding author upon request. The data that support the findings of this study are available upon request from the corresponding author.

## References

[1] Serjeant G.R. (1997). Sickle-cell disease. Lancet.

[2] Elendu C., Amaechi D.C., Alakwe-Ojimba C.E., Elendu T.C., Elendu R.C., Ayabazu C.P., Aina T.O., Aborisade O., Adenikinju J.S. (2023). Understanding Sickle Cell Disease: Causes, Symptoms, and Treatment Options. Medicine.

[3] Tebbi C.K. (2022). Sickle Cell Disease, a Review. Hemato.

[4] Ershler W.B., De Castro L.M., Pakbaz Z., Moynahan A., Weycker D., Delea T.E., Agodoa I., Cong Z. (2023). Hemoglobin and End-Organ Damage in Individuals with Sickle Cell Disease. Curr. Ther. Res..

[5] Mehta A.B., Hoffbrand A.V. (2000). Hemolytic Anaemias V Inherited Defects of Haemoglobin—Sickle Cell Disease. Haematology at a Glance.

[6] Quinn C.T. (2016). Minireview. Clinical Severity in Sickle Cell Disease: The Challenges of Definition and Prognostication. Exp. Biol. Med..

[7] Inusa B.P.D., Hsu L.L., Kohli N., Patel A., Ominu-Evbota K., Anie K.A., Atoyebi W. (2019). Sickle Cell Disease—Genetics, Pathophysiology, Clinical Presentation and Treatment. Int. J. Neonatal Screen..

[8] Kargutkar N., Sawant-Mulay M., Hariharan P., Chandrakala S., Nadkarni A. (2023). Role of MicroRNA in Hydroxyurea Mediated HbF Induction in Sickle Cell Anaemia Patients. Sci. Rep..

[9] Stuart M.J., Nagel R.L. (2004). Sickle-Cell Disease. Lancet.

[10] Habara A., Steinberg M.H. (2015). Minireview: Genetic Basis of Heterogeneity and Severity in Sickle Cell Disease. Exp. Biol. Med..

[11] Du M., Van Ness S., Gordeuk V., Nouraie S.M., Nekhai S., Gladwin M., Steinberg M.H., Sebastiani P. (2018). Biomarker Signatures of Sickle Cell Disease Severity. Blood Cells Mol. Dis..

[12] Njoku F., Zhang X., Shah B.N., Machado R.F., Han J., Saraf S.L., Gordeuk V.R. (2021). Biomarkers of Clinical Severity in Treated andUntreated Sickle Cell Disease: A Comparison by Genotypes of a Single Center Cohort and African Americans in the NHANESStudy. Br. J. Haematol..

[13] Hazzazi A., Ageeli A., Alfaqih M.H., Ali A.M., Jaafari A., Malhan H.M., Bakkar M.M. (2020). Epidemiology and Characteristics of Sickle Cell Patients Admitted to Hospitals in Jazan Region. Saudi Arabia. J. Appl. Hematol..

[14] Arji E.E., Eze U.J., Ezenwaka G.O., Kennedy N. (2023). Evidence-Based Interventions for Reducing Sickle Cell Disease-Associated Morbidity and Mortality in Sub-Saharan Africa: A Scoping Review. SAGE Open Med..

[15] Piel F.B., Patil A.P., Howes R.E., Nyangiri O.A., Gething P.W., Dewi M., Temperley W.H., Williams T.N., Weatherall D.J., Hay S.I. (2013). Global Epidemiology of Sickle Haemoglobin in Neonates: A Contemporary Geostatistical Model-Based Map and Population Estimates. Lancet.

[16] Sedrak A., Kondamudi N.P. (2003). Sickle Cell Disease. StatPearls [Internet].

[17] Jastaniah W. (2011). Epidemiology of sickle cell disease in Saudi Arabia. Ann. Saudi Med..

[18] Kato G.J., Piel F.B., Reid C.D., Gaston M.H., Ohene-Frempong K., Krishnamurti L., Smith W.R., Panepinto J.A., Weatherall D.J., Costa F.F. (2018). Sickle cell disease. Nat. Rev. Dis. Primers.

[19] Modell B., Darlison M. (2008). Global epidemiology of haemoglobin disorders and derived service indicators. Bull. World Health Organ..

[20] World Health Organization Management of haemoglobin disorders. Proceedings of the Report of Joint WHO-TIF Meeting.

[21] Deo P.N., Deshmukh R. (2019). Oral microbiome: Unveiling the fundamentals. J. Oral Maxillofac. Pathol..

[22] Lamont R.J., Koo H., Hajishengallis G. (2018). The oral microbiota: Dynamic communities and host interactions. Nat. Rev. Microbiol..

[23] Thuy D., Devine D., Marsh P. (2013). Oral biofilms: Molecular analysis, challenges, and future prospects in dental diagnostics. Clin. Cosm. Investig. Dent..

[24] Marsh P.D., Zaura E. (2017). Dental biofilm: Ecological interactions in health and disease. J. Clin. Periodontol..

[25] de Carvalho H.L.C.C., Rolim J.Y.S., Thomaz E.B.A.F., Souza S.d.F.C. (2017). Are dental and jaw changes more prevalent in a Brazilian population with sickle cell anemia. Oral Surg. Oral Med. Oral Pathol. Oral Radiol..

[26] Taylor L.B., Nowak A.J., Giller R.H., Casamassimo P.S. (1995). Sickle cell anemia: A review of the dental concerns and a retrospective study of dental and bony changes. Spec. Care Dent..

[27] Souza S., de Carvalho H., Costa C., Thomaz E. (2018). Association of sickle cell haemoglobinopathies with dental and jaw bone abnormalities. Oral Dis..

[28] Rada R.E., Bronny A.T., Hasiakos P.S. (1987). Sickle cell crisis precipitated by periodontal infection: Report of two cases. J. Am. Dent. Assoc..

[29] Laurence B., George D., Woods D., Shosanya A., Katz R.V., Lanzkron S., Diener-West M., Powe N. (2006). The association between sickle cell disease and dental caries in African Americans. Spec. Care Dent..

[30] Singh J., Singh N., Kumar A., Kedia N.B., Agarwal A. (2013). Dental and periodontal health status of Beta thalassemia major and sickle cell anemic patients: A comparative study. J. Int. Oral Health.

[31] Al-Alawi H., Al-Jawad A., Al-Shayeb M., Al-Ali A., Al-Khalifa K. (2015). The association between dental and periodontal diseases and sickle cell disease. A pilot case-control study. Saudi Dent. J..

[32] Helaly M., Abuaffan A. (2015). Association between sickle cell disease and dental caries among Sudanese children. J. Mol. Imag. Dynamic..

[33] Brandão C.F., Oliveira V.M.B., Santos A.R.R.M., da Silva T.M.M., Vilella V.Q.C., Simas G.G.P.P., Carvalho L.R.S., Carvalho R.A.C., Ladeia A.M.T. (2018). Association between sickle cell disease and the oral health condition of children and adolescents. BMC Oral Health.

[34] Pitts N., Zero D. (2016). White paper on dental caries prevention and management: A summary of the current evidence and the key issues in controlling this preventable disease. FDI World Dent. Fed..

[35] Pitts N.B., Zero D.T., Marsh P.D., Ekstrand K., Weintraub J.A., Ramos-Gomez F., Tagami J., Twetman S., Tsakos G., Ismail A. (2017). Dental caries. Nat. Rev. Dis. Primers.

[36] Sampaio-Maia B., Caldas I.M., Pereira M.L., Perez-Mongiovi D., Araujo R. (2016). The oral microbiome in health and its implication in oral and systemic diseases. Adv. Appl. Microbiol..

[37] Amir A., McDonald D., Navas-Molina J.A., Kopylova E., Morton J.T., Zech Xu Z., Kightley E.P., Thompson L.R., Hyde E.R., Gonzalez A. (2017). Deblur rapidly resolves single-nucleotide community sequence patterns. MSystems.

[38] McMurdie P.J., Holmes S. (2013). Phyloseq: An R package for reproducible interactive analysis and graphics of microbiome census data. PLoS ONE.

[39] Alyousef Y.M., Alonaizan F.A., Alsulaiman A.A., Aldarwish M.I., Alali A.A., Almasood N.N., Vatte C., Cyrus C., Habara A.H., Koeleman B.P.C. (2023). Oral microbiota analyses of Saudi sickle cell anemics with dental caries. Int. Dent. J..

[40] Rizzardi K.F., Indiani C.M., dos S.P., Mattos-Graner R.d.O., de Sousa E.T., Nobre-dos-Santos M., Parisotto T.M. (2021). Firmicutes Levels in the Mouth Reflect the Gut Condition with Respect to Obesity and Early Childhood Caries. Front. Cell. Infect. Microbiol..

[41] Dewhirst F.E., Chen T., Izard J., Paster B.J., Tanner A.C.R., Yu W.H., Lakshmanan A., Wade W.G. (2010). The Human Oral Microbiome. J. Bacteriol..

[42] Xiao J., Huang X., Alkhers N., Alzamil H., Alzoubi S., Wu T.T., Castillo D.A., Campbell F., Davis J., Herzog K. (2018). Candida albicans and Early Childhood Caries: A Systematic Review and Meta-Analysis. Caries Res..

[43] Abranches J., Zeng L., Kajfasz J., Palmer S., Chakraborty B., Wen Z., Richards V., Brady L., Lemos J. (2018). Biology of Oral Streptococci. Microbiol. Spect..

[44] Patini R., Staderini E., Lajolo C., Lopetuso L., Mohammed H., Rimondini L., Rocchetti V., Franceschi F., Cordaro M., Gallenz P. (2018). Relationship between oral microbiota and periodontal disease: A systematic review. Eur. Rev. Med. Pharmaco. Sci..

[45] Nomura Y., Otsuka R., Hasegawa R., Hanada N. (2020). Oral Microbiome of Children Living in an Isolated Area in Myanmar. Int. J. Environ. Res. Public Health.

[46] Yue H., Xu X., Liu Q., Li X., Jiang W., Hu B. (2020). Association between sickle cell disease and dental caries: A systematic review and meta-analysis. Hematology.

[47] Könönen E., Gursoy U.K. (2022). Oral Prevotella Species and Their Connection to Events of Clinical Relevance in Gastrointestinal and Respiratory Tracts. Front. Microbiol..

